# The long arm of childhood socioeconomic deprivation on mid‐ to later‐life cognitive trajectories: A cross‐cohort analysis

**DOI:** 10.1002/dad2.12322

**Published:** 2022-06-01

**Authors:** Ruby S. M. Tsang, John E. Gallacher, Sarah Bauermeister

**Affiliations:** ^1^ Department of Psychiatry University of Oxford Warneford Hospital Oxford UK

**Keywords:** childhood socioeconomic status, cognitive aging, cognitive decline, latent class mixed models, longitudinal studies

## Abstract

**Introduction:**

Earlier studies of the effects of childhood socioeconomic status (SES) on later‐life cognitive function consistently report a social gradient in later‐life cognitive function. Evidence for their effects on cognitive decline is, however, less clear.

**Methods:**

The sample consists of 5324 participants in the Whitehall II study, 8572 in the Health and Retirement Study (HRS), and 1413 in the Kame Project, who completed self‐report questionnaires on their early life experiences and underwent repeated cognitive assessments. We characterized cognitive trajectories using latent class mixed models, and explored associations between childhood SES and latent class membership using logistic regressions.

**Results:**

We identified distinct trajectories classes for all cognitive measures examined. Childhood socioeconomic deprivation was associated with an increased likelihood of being in a lower trajectory class.

**Discussion:**

Our findings support the notions that cognitive aging is a heterogeneous process and early life circumstances may have lasting effects on cognition across the life‐course.

## 1 INTRODUCTION

Childhood adversity is known to have profound effects on cognitive development,^1^ with cognitive deficits or delays reported in childhood and adolescence among those who were exposed to childhood adversity.^2^ Current conceptualization of the neurodevelopmental effects of childhood adversity suggests that exposure to childhood adversity results in the dysregulation of the hypothalamic‐pituitary‐adrenal (HPA) axis. The HPA axis is activated and glucocorticoids are released in the face of stressful experiences. With childhood adversity, the brain is exposed to prolonged periods of excessive glucocorticoid release during sensitive periods of development, which may result in lasting structural and functional changes in the brain. In addition to the HPA axis, other mechanisms such as the immune system, the microbiome, as well as epigenetic alterations may also play a role in the detrimental health effects linked to child adversity.^3^, ^4^

Given that later life cognitive function is determined to a great extent by childhood cognition,^5^ it has been hypothesized that the impact of childhood adversity may persist into later life, and one of the most frequently studied form of childhood adversity in aging studies is childhood socioeconomic deprivation. Studies consistently report a social gradient in absolute later‐life cognitive function, with lower childhood socioeconomic status (SES) associated with poorer global cognition,^6^, ^7^, ^8^, ^9^, ^10^, ^11^, ^12^, ^13^ memory,^11^, ^12^, ^14^, ^15^, ^16^, ^17^, ^18^ verbal fluency,^17^, ^18^ language,^11^ processing speed,^11^, ^16^ visuospatial abilities,^11^ and executive function^12^ in mid to later life.

However, the literature on whether childhood SES is associated with cognitive decline is largely inconsistent. Although the majority of studies did not find an association,^6^, ^7^, ^9^, ^10^, ^11^, ^13^, ^15^, ^16^, ^17^, ^19^, ^20^ the few exceptions reported associations in opposite directions. For instance, one study reported that higher childhood SES was associated with slower global cognitive decline, but not with decline in specific cognitive components (episodic memory, semantic memory, and executive function),^14^, ^21^ whereas another found that higher childhood SES was associated with faster cognitive decline.^18^ Other factors such as race or sex may also modify the association. Although being very poor or having poor health in childhood was not associated with faster cognitive decline, not having enough food to eat and being thinner than average in childhood were associated with slower global cognitive decline among African Americans; these effects were not observed in Caucasians.^8^ Furthermore, men in the middle childhood SES group showed faster decline in processing speed, whereas women in the low childhood SES group showed slower decline in memory and global cognition.^12^ These findings are counterintuitive. The proposition that that early adversity lowers cognitive performance, but either does not affect or improves (slows) cognitive decline, deserves further attention.

Studies exploring the relationships between childhood SES and cognitive decline typically use mixed‐effects or growth‐curve models, both of which estimate an overall mean trajectory for the entire sample, or sub‐sample, and individual variation around this mean trajectory.^22^ However, more recently, there is increasing evidence to suggest that cognitive aging is a heterogeneous process and that distinct subgroups of trajectories exist between individuals and across cognitive domains.^23^, ^24^ For this reason, the use of mixed‐effects or growth‐curve models may not be the most appropriate method for modeling cognitive decline and its relationship with childhood SES.

**RESEARCH IN CONTEXT**

1. **Systematic Review**: We reviewed the literature on childhood socioeconomic status (SES) as a predictor for cognitive decline in mid to later life using PubMed. Studies generally reported that lower childhood SES is associated with poorer baseline cognition, but not a faster rate of decline. These studies generally focused on the mean rate of decline in the population; no study to date has explored associations between childhood SES and different cognitive trajectories. Relevant studies have been cited appropriately.
2. **Interpretation**: Our findings suggest that cognitive trajectories differ between individuals and across cognitive domains. Individuals of lower childhood SES were more likely to be in a lower cognitive trajectory class, which may or may not involve more rapid decline.
3. **Future Directions**: Future studies should include more cognitive outcomes and longer follow‐ups, as well as investigate the impact of social mobility to further improve our understanding of how early life circumstances influence cognitive decline.

**HIGHLIGHTS**

- Studies consistently report a social gradient in later life cognitive function.
- Effects of childhood socioeconomic deprivation on cognitive decline is less clear.
- We identified distinct cognitive trajectories for all cognitive domains examined.
- Those from a deprived childhood were more likely to be in a lower trajectory class.

The aim of this study is to obtain further insights into the relationship between childhood SES and cognitive decline in mid to later life. We seek to first identify latent classes of cognitive trajectories, and then to examine the predictive utility of childhood SES indicators on class membership using secondary data from three aging cohorts, namely the Whitehall II study,^25^ the Health and Retirement Study (HRS),^26^ and the Kame Project.^27^

## 2 METHODS

### 2.1 Cohort and study sample selection

Cohort selection was undertaken using the Dementias Platform UK (DPUK) Data Discovery tools. Our inclusion criteria were studies with:

1. Participants 50 years of age and older;
2. Cognitive data from three or more assessment points using the same instrument(s);
3. Childhood SES data; and
4. The data were already available to access on the DPUK Data Portal^28^ at the time of the study.

We then investigated whether data from cohorts on other platforms may be appropriate for this study, and the relevant data were uploaded to the DPUK Data Portal with permission.

Few aging cohorts have collected data on both cognition and childhood adversity; following the aforementioned selection procedure, the Whitehall II study, the HRS, and the Kame Project were included in this study as they were the only ones that met the requirements of our study. Although the measures used in these three cohorts are different, we believe this is a reflection of the cross‐cultural nature of this study. A brief overview of each of these cohorts are provided below.

Whitehall II study: The Whitehall II study is a cohort of 10 308 British civil servants (3413 women and 6895 men) 35 to 55 years in 1985 to 1988, with the aim of investigating social inequalities in health. These participants were recruited from different employment grades, and over the years, some may have left the civil service and many are now retired. It began with full‐cohort follow‐ups in the research clinic occurring at 5‐year intervals, involving clinical assessments and self‐report questionnaires, and a postal questionnaire is sent to participants in‐between clinic phases.^25^ Data collected include sociodemographic characteristics, health behaviors, questionnaires about physical and mental health, anthropometric measurements, blood samples, and neuropsychological test performance.

HRS: The HRS is a longitudinal panel study of a representative sample of individuals in the United States. Community‐dwelling adults born between 1931 and 1941 (ages 51–61 years at the time) sampled at the household level were recruited into the initial cohort in 1992. It was subsequently merged with the Asset and Health Dynamics Among the Oldest Old (AHEAD; born between 1890 and 1923), the Children of the Depression (born between 1924 and 1930), and the War Babies (born between 1942 and 1947) cohorts to be fully representative of the US older population. Data collected include sociodemographic characteristics, medical history, anthropometric measures, functional limitations, questionnaires about family and relationships, and neuropsychological test performance.

Kame Project: The Kame Project is a population‐based prospective study of aging and dementia of 1985 community‐ and institution‐dwelling Japanese Americans 65 years of age and older residing in King County, WA. The study began in 1992, and follow‐ups were carried out at ≈2, 4, 6, and 8 years. Data collected included sociodemographic characteristics, medical history, health behaviors, anthropometric measurements, apolipoprotein E (*APOE*) genotype status, and neuropsychological test performance.

Participants with data in at least half of the selected data collection waves were included in the analyses. Because only those equal to or older than 65 years in HRS and those older than 60 years in the Kame Project completed the cognitive tests of interest, samples were restricted to those who were older than these respective age cut‐offs. Participants in all three cohorts provided written informed consent at the time of data collection.

### 2.2 Cognitive outcomes

In the Whitehall II study, cognitive data were taken from phases 7, 9, 11, and 12 (which took place in 2002–2004, 2007–2009, 2012–2013, and 2015–2016, respectively). Global cognition was assessed with the Mini–Mental State Examination (MMSE),^29^ verbal memory with a 20‐word free recall, and fluency with a 60‐second written naming task of words beginning with the letter "S." In HRS, cognitive data were obtained from years 2010 to 2016. Global cognition was assessed with a modified version of the Telephone Interview for Cognitive Status (TICS‐M),^30^ which includes items that assess memory, attention, orientation and language, and fluency with a 60‐second verbal animal naming task. In the Kame Project, cognitive data were from the first five visits (1992–1994, 1994–1996, 1996–1998, 1998–2000, and 2000–2001), and global cognition was assessed using the MMSE.

### 2.3 Childhood SES indicators

Participants in the three cohorts completed self‐report questionnaires that included items that reflect childhood SES. The items varied between the cohorts, but covered aspects including parental education, parental unemployment, and family financial hardship. The full list of these items and details on the variable coding are presented in Table A1 in the Supplementary Appendix.

### 2.4 Covariates

Covariates that were tested in the models include age at the selected baseline, sex, and years of education.

### 2.5 Statistical analyses

Latent class mixed models were used to identify subgroups of participants with similar cognitive performance across time. This was performed using the *lcmm* package version 1.7.8^31^ in R version 3.5.3. In these models, a latent class model is used to identify latent subgroups of individuals based on their trajectories and a mixed model is used to describe the mean trajectory within each subgroup simultaneously. The main underlying assumption is that the population is heterogeneous and composed of multiple latent classes with their own respective mean profiles of trajectories.

These models attempt to explain the dependent variable (cognition in this case) as a function of time at the population level (fixed component), the class‐specific level (mixture component), and the individual level (random component).

We used a data‐driven approach adapted from methods used by Carrière et al.^32^ All models used a beta cumulative distribution function transformation to address skewness in the data. We first estimated the cognitive trajectories without adjustment for baseline covariates, then sequentially increased model complexity (intercept‐only, linear, or quadratic time effects for fixed, mixture, and random effects, and from one to three latent classes). For each cognitive outcome, a total of 34 models were tested (see Table A2 in the Supplementary Appendix). Goodness‐of‐fit was assessed based on model convergence, Bayesian information criterion (BIC), and average posterior probabilities (AvePP). Lower estimates of BIC indicate better model fit, and AvePP >0.7 for all trajectory classes indicate high accuracy in class assignment.^33^ Then, covariates were introduced into the class‐membership model in separate baseline age‐adjusted models, and those with a *P*‐value <.20 were included in the final model. Where the final model either failed to converge or returned a smallest class being <1% of the sample, the model with the next lowest BIC value and AvePP >0.7 for all identified classes would be tested for covariates, and so forth.

After participants were classified into subgroups, logistic regressions were carried out separately with each childhood SES indicator as the predictor of class membership in Stata/SE 15.1. We accounted for multiple comparisons using the Benjamini‐Hochberg procedure,^34^ with the false discovery rate controlled at 0.05.

All analyses were carried out in the DPUK Data Portal.^28^

## 3 RESULTS

Descriptive statistics of the included participants from the three cohorts are presented in Table 1.

**TABLE 1:** Sample descriptives at the selected baseline

Variable	Baseline used in this study	Follow‐up 1	Follow‐up 2	Follow‐up 3	Follow‐up 4
**Whitehall II study (*n* = 5324)**	**Phase 7**	**Phase 9**	**Phase 11**	**Phase 12**	
Age at questionnaire	50–54: 1034 (19.4%) 55–59: 1644 (30.9%) 60–64: 1139 (21.4%) 65–69: 1062 (19.9%) 70–74: 445 (8.4%)	Missing: 63 55–59: 1017 60–64: 1621 65–69: 1113 70–74: 1058 75–79: 452	Missing: 312 55–59: 8 60–64: 1270 65–69: 1499 70–74: 1046 75–79: 932 80–84: 257	Missing: 741 60–64: 292 65–69: 1562 70–74: 1177 75–79: 862 80–84: 659 85–89: 31	
Age at screening	Missing: 78 (1.5%) 50–54: 1015 (19.1%) 55–59: 1618 (30.4%) 60–64: 1121 (21.1%) 65–69: 1050 (19.7%) 70–74: 442 (8.3%)	Missing: 99 (1.9%) 55–59: 1007 (18.9%) 60–64: 1610 (30.2%) 65–69: 1110 (20.8%) 70–74: 1048 (19.7%) 75–79: 450 (8.5%)	59–64: 1750 (32.9%) 65–69: 1455 (27.3%) 70–74: 1001 (18.8%) 75–79: 879 (16.5%) 80–83: 239 (4.5%)	Missing: 1034 (19.4%) 62–64: 274 (5.1%) 65–69: 1495 (28.1%) 70–74: 1111 (20.9%) 75–79: 790 (14.8%) 80–85: 592 (11.1%) 85+: 28 (0.5%)	
Sex	M: 3875 (72.8%) F: 1449 (27.2%)				
Education (years)	15.09 ± 4.15				
Mini‐Mental State Examination (MMSE)	28.77 ± 1.21	28.51 ± 1.22	28.34 ± 1.62	28.36 ± 1.72	
Phonemic fluency	15.95 ± 4.08	15.43 ± 3.95	15.25 ± 4.25	14.87 ± 4.62	
Memory	6.90 ± 2.35	6.24 ± 2.21	6.05 ± 2.37	5.35 ± 2.19	
Time since last wave (years)		4.98 ± 0.37	4.13 ± 0.45	3.19 ± 0.52	
**Health and Retirement Study (HRS) (*n* = 8572)**	**2010**	**2012**	**2014**	**2016**	
Age	74.70 ± 6.64	76.44 ± 6.62	77.95 ± 6.43	79.51 ± 6.02	
Sex	M: 3590 (41.88%) F: 4982 (58.12%)				
Education (years)	12.50 ± 3.16				
Modified Telephone Interview for Cognitive Status (TICS‐M)	21.64 ± 4.87	21.02 ± 5.26	20.87 ± 5.40	20.66 ± 5.43	
Semantic fluency	15.15 ± 6.50	15.19 ± 6.46	15.16 ± 6.35	15.15 ± 6.34	
Time since last wave (months)		20.93 ± 3.82	22.44 ± 2.80	26.57 ± 3.80	
**Kame Project** (*n* = 1413)	Visit 0	Visit 1	Visit 2	Visit 3	Visit 4
Age	70.95 ± 4.83	72.92 ± 4.86	74.97 ± 4.90	76.82 ± 4.73	78.31 ± 4.66
Sex	M: 615 (43.5%) F: 798 (56.5%)				
Education (years)	13.17 ± 2.80				
Mini‐Mental State Examination (MMSE)	26.51 ± 2.29	26.87 ± 2.16	26.70 ± 2.45	26.64 ± 2.38	26.63 ± 2.55
Time since last wave (years)		1.98 ± 0.32	2.04 ± 0.44	2.10 ± 0.30	1.80 ± 0.28

*Note*: *n* (%) or mean ± SD.

The granularity of data on interview date that we have access to differs for the three cohorts; the Whitehall II Study provides only calendar year; the Health and Retirement Study, the year and month; and the Kame Project, the year, month, and date. The time between waves for the Whitehall II Study is a crude estimate based on calendar year data.

### 3.1 Characterization of cognitive trajectories

#### 3.1.1 Whitehall II study

**FIGURE 1:**
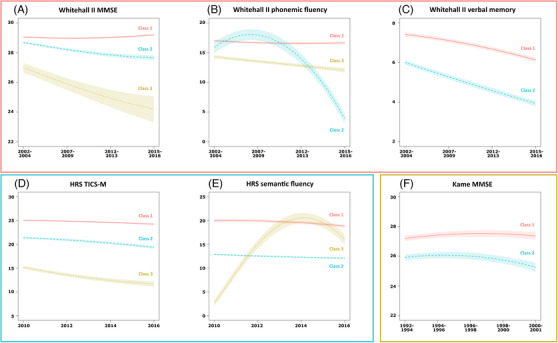
Mean predicted trajectories for the identified classes in the Whitehall II Study: (A) Mini‐Mental State Examination (MMSE), (B) phonemic fluency, (C) memory; in the Health and Retirement Study (HRS): (D) Modified Telephone Interview for Cognitive Status (TICS‐M), (E) semantic fluency; and in the Kame Project: (F) Mini‐Mental State Examination (MMSE).

In the Whitehall II study, the best‐fitting model for all three cognitive measures showed quadratic decline in the fixed and mixture components; however, they differed in the random component where there was no decline in global cognition but linear decline in fluency and memory (Table 2). The patterns of trajectories appeared to be different across cognitive domains. Three classes of trajectory were identified for global cognition and for fluency, and two for memory. For global cognition the classes correspond to a stable trajectory, a gradual decline trajectory, and a relatively rapid decline trajectory (Figure 1A). For fluency, the three classes represent a stable trajectory, a gradual decline trajectory, and a curvilinear trajectory showing an initial improvement followed by rapid decline (Figure 1B). Both memory classes showed decline over time (Figure 1C).

**TABLE 2:** Parameters, model fit indices, and class assignment in the final latent class mixed models

	Fixed effects	Mixture	Random effects	Covariates	No. of latent classes	Class assignment	BIC
Whitehall II study							
Mini‐Mental State Examination (MMSE)	*β_0_* + *β_1_* T + *β_2_* T^2^	*α_0k_* + *α_1k_* T + *α_2k_* T^2^	*u_0ki_*	Age, sex, education	3	2729 (51.26%) 2407 (45.21%) 188 (3.53%)	56256.96
Phonemic fluency	*β_0_* + *β_1_* T + *β_2_* T^2^	*α_0k_* + *α_1k_* T + *α_2k_* T^2^	*u_0ki_* + *u_1ki_* T	Age, sex, education	3	3192 (59.95%) 97 (1.82%) 2035 (38.22%)	98964.64
Memory	*β_0_* + *β_1_* T + *β_2_* T^2^	*α_0k_* + *α_1k_* T + *α_2k_* T^2^	*u_0ki_* + *u_1ki_* T	Age, education	2	3219 (60.46%) 2105 (39.54%)	79919.54
HRS							
Modified Telephone Interview for Cognitive Status (TICS‐M)	*β_0_* + *β_1_* T + *β_2_* T^2^	*α_0k_* + *α_1k_* T + *α_2k_* T^2^	*u_0ki_* + *u_1ki_* T	Age, sex, education	3	2789 (32.54%) 4420 (51.56%) 1363 (15.90%)	160218.94
Semantic fluency	*β_0_* + *β_1_* T + *β_2_* T^2^	*α_0k_* + *α_1k_* T + *α_2k_* T^2^	*u_0ki_* + *u_1ki_* T	Age, sex, education	3	2847 (33.21%) 5591 (65.22%) 134 (1.56%)	182945.71
Kame Project							
Mini‐Mental State Examination (MMSE)	*β_0_* + *β_1_* T + *β_2_* T^2^	*α_0k_* + *α_1k_* T + *α_2k_* T^2^	*u_0ki_* + *u_1ki_* T	Age, sex, education	2	706 (49.96%) 707 (50.04%)	26646.39

#### 3.1.2 Health and retirement study

The best‐fitting model for both global cognition and fluency in HRS showed quadratic decline in the fixed and mixture components, and linear decline in the random component (Table 2). Both models identified three trajectories. For global cognition all showed gradual decline with suggested different baseline levels (Figure 1D), whereas for fluency although two trajectories showed gradual decline, the third showed initial improvement followed by rapid decline (Figure 1E).

#### 3.1.3 Kame Project

In the Kame Project, global cognition showed quadratic decline in the fixed and mixture components, and linear decline in the random component. The two curves correspond to a stable trajectory and a gradual decline trajectory (Figure 1F).

### 3.2 Associations between exposure to early adversity and class membership

Using Class 1 as the reference group, it appeared that among the childhood SES indicators examined, almost all showed an association between lower childhood SES and increased likelihood of membership in a lower trajectory class (Table 3).

**TABLE 3:** Associations between childhood SES indicators and the likelihood of being in a lower trajectory (with the top trajectory “Class 1” as reference)

	Global cognition Class 2		Global cognition Class 3		Fluency Class 2		Fluency Class 3		Memory Class 2	
Childhood SES indicator	*B*	*P*	*B*	*P*	*B*	*P*	*B*	*P*	*B*	*P*
Whitehall II study										
Age when father finished full‐time education	−0.06*	<.0001	−0.05	.2392	−0.03	.5568	−0.10*	<.0001	−0.08*	<.0001
Age when mother finished full‐time education	−0.11*	<.0001	−0.26*	.0001	−0.11	.1057	−0.18*	<.0001	−0.14*	<.0001
Father's social class	−0.11*	.0001	−0.24*	.0017	−0.12	.2589	−0.16*	<.0001	−0.10*	.0003
Spent four or more weeks in hospital	0.14	.1126	0.13	.5800	0.46	.1098	0.38*	<.0001	0.35*	<.0001
Father/mother were unemployed when they wanted to be working	0.31*	.0010	0.59*	.0082	−0.68	.1420	0.13	.1629	0.27*	.0031
Family had continuing financial problems	0.17*	.0073	0.36*	.0309	0.15	.5305	0.29*	<.0001	0.31*	<.0001
Family/household did not have an inside toilet	0.36*	<.0001	0.74*	<.0001	0.40	.0973	0.50*	<.0001	0.43*	<.0001
Family/household owned a car	−0.60*	<.0001	−1.28*	<.0001	−0.65*	.0028	−0.97*	<.0001	−0.95*	<.0001
HRS										
Father's education	−0.12*	<.0001	−0.27*	<.0001	−0.14*	<.0001	−0.07	.1023		
Mother's education	−0.14*	<.0001	−0.32*	<.0001	−0.16*	<.0001	−0.14*	.0009		
Childhood health	−0.20*	<.0001	−0.45*	<.0001	−0.24*	<.0001	−0.08	.3850		
Family financially poor	0.50*	<.0001	0.82*	<.0001	0.48*	<.0001	0.32	.0881		
Family moved due to financial difficulties	0.30*	<.0001	0.33*	.0001	0.14*	.0210	0.29	.1856		
Family received help because of financial difficulties	−0.01	.8708	−0.19	.0745	−0.20*	.0036	−0.07	.7791		
Father unemployed	0.32*	<.0001	0.37*	<.0001	0.28*	<.0001	0.25	.2458		
Kame Project										
Father's education	−0.09*	<.0001								
Mother's education	−0.10*	<.0001								
Household density	0.07	.1734								
Urban/suburban living	−0.41*	.0003								
Family financial difficulties	0.04	.0556								

^*^FDR < 0.05.

In the Whitehall II study, older age when father completed full‐time education, older age when mother completed full‐time education, higher father's social class, and family car ownership were associated consistently with a decreased likelihood of a lower trajectory across cognitive domains, whereas ongoing family financial problems and not having an inside toilet in the household were associated with a greater likelihood of being in a lower cognitive trajectory. Having spent four or more weeks in hospital and parental unemployment were associated with a greater likelihood of being in a lower trajectory for two of three cognitive outcomes. For fluency there was limited evidence for an association between childhood SES and initial learning effects, although rapid subsequent decline was strongly indicated.

Similarly, almost all childhood SES indicators examined were associated with an increased likelihood of membership in a lower cognitive trajectory in HRS. Higher father's education, higher mother's education, better childhood health were consistently associated with a decreased likelihood of a lower trajectory. Worse family financial status, family having moved due to financial difficulties, and father's unemployment were related to greater likelihood of a lower trajectory. For fluency, in comparison with Whitehall II, the evidence for an association between childhood SES and initial learning effects was stronger, whereas evidence for rapid decline was weaker.

The results from the Kame Project, however, present a mixed picture. On one hand, higher father's education, higher mother's education, and urban/suburban living were linked to a lower likelihood of a lower cognitive trajectory. On the other hand, household density and family financial situation were not associated with the likelihood of being in a lower trajectory.

## 4 DISCUSSION

Using longitudinal data from three population cohorts, cognitive trajectory was found to be associated with childhood adversity. Latent class mixed modeling found that membership of more rapidly declining groups was associated with a variety of adversity indicators including childhood SES, parental education, and financial problems. This indicates that childhood adversity affects cognitive decline and not just cognitive performance.

For global cognition, using two measures (MMSE and TICS‐M) three trajectories were identified for the Whitehall II study and HRS, and two for the Kame Project. That membership of faster declining groups was associated with a range of early adversity indicators is not surprising because a measure of global cognition will capture to a limited extent a range of domain‐specific effects.

For verbal and semantic fluency in the Whitehall II study and HRS, although the fluency tasks were slightly different, there was a small group that showed a strongly curvilinear trajectory, suggesting improved performance followed by rapid decline. This could be an artifact of the test, that is, a practice effect, or it could be instability in the analysis due to small group size. That it was found in both the Whitehall II study and HRS may be considered surprising for a statistical artifact. Practice effects have been found even when assessments were conducted several years apart, and such short‐term improvements are often large enough to counteract age‐related cognitive decline.^35^ Although it may seem counterintuitive that those who will eventually be cognitively impaired in fact show greater practice effects, one explanation is that these individuals were performing below their actual cognitive potential when they first encounter novel cognitive tests, as they need more time to understand the task demands. As they familiarize themselves with the task characteristics, they then exhibit a “rebound” in their performance (i.e., a “novelty effect”).^36^, ^37^ Thorgusen and colleagues^38^ demonstrated that both memory and novelty uniquely contribute toward practice effects, and cognitive impairment is more likely to be associated with smaller practice effects in memory tasks, but larger practice effects in tasks assessing other cognitive domains. It has been proposed that such novelty effect may be a useful early marker of declining cognitive reserve and neurodegeneration, and that this would be consistent with the shape of the curves found here. Nevertheless, further work is required before conclusions can be drawn with confidence.

For memory, the association with group membership was found for all indicators of early adversity. Stronger associations were found for what might be described as dynamic indicators (those reflecting episodes of adversity), rather than contextual indicators (those reflecting demographic strata such as SES). Although this is also found for fluency and global cognition, it is most clearly demonstrated for memory. This is a potentially important observation, suggesting that it is the greater likelihood of specific events (psycho‐social insults) that come with lower social status that have the greater cognitive impact.

Nevertheless, our results suggest that childhood SES is an important contributor to mid‐ to later‐life cognitive decline. Level of education is often used as a measure of early life SES; there is consistent evidence that education plays an important role in later‐life cognitive function and cognitive decline,^39^, ^40^, ^41^ but few studies have examined the effects of other measures of childhood SES, especially on cognitive decline. This article adds to the literature by including a range of childhood SES indicators, and examining their associations with cognitive trajectories.

The strengths of this study include the comparison populations and cognitive measures, and the use of latent trajectories to more clearly model change. Although distinct classes of trajectory were identified, differences in slope between classes varied across studies. In the Whitehall II study, differences in slope between trajectories were more pronounced than in HRS or the Kame Project. Although it is possible that adversity does not differentially affect the rate of cognitive decline in the HRS and Kame Project populations, it is unlikely that comparable exposures would differentially affect comparable outcomes. More likely this is due to the Whitehall II study providing more variation with which to test the hypothesis due to having 12 years of follow‐up, compared to the 6 years of HRS and 8 years of the Kame Project. Nevertheless, that differences in slope vary may explain why some previous studies using less‐sensitive techniques report no association between childhood SES and rate of cognitive decline.^6^, ^7^, ^9^, ^10^, ^11^, ^13^, ^15^, ^16^, ^17^, ^19^, ^20^ However, two studies report less decline with early adversity. One reports on a small and highly diverse cohort over a 5‐year follow‐up period.^14^, ^21^ The other describes a relatively large and representative sample followed for 12 years.^18^ It is concluded that faster decline in high‐performing groups (those with less early adversity) may be due to greater cognitive reserve delaying the inflexion point for decline, but once the inflexion point has been passed, it is followed by rapid decline. It would be interesting to test this hypothesis using a latent class mixed‐modeling approach, which is better suited to detect the effect of an inflexion point. From a cognitive reserve perspective, our data suggest that early adversity is associated with earlier inflexion.

Some theoretical and methodological issues should be addressed. First, observable cognitive change across time is partly dependent on the psychometric properties of the cognitive instruments used. For instance, MMSE is known to have a strong ceiling effect^42^ and shows poor sensitivity to change in the upper tail of the distribution.^43^, ^44^ The curvilinear nature of the instrument means that a one‐point change in the higher range of scores does not hold the same clinical meaning as a one‐point change in the medium or lower range. This ceiling effect, in combination with common practice or retest effects, could lead to biased estimates of cognitive change and potentially spurious latent trajectories.^45^ Practice effects may also differ by background characteristics, which could lead to biased associations between childhood SES and cognitive aging trajectories.^46^

Second, the magnitude of cognitive change observed is somewhat dependent on the length and frequency of follow‐up, as well as the demographic characteristics of the sample. Differences in follow‐up period have been described earlier. The cohorts also differ demographically. Although the Whitehall II study is a cohort of British civil servants, the HRS is a representative sample of US citizens, whereas the Kame Project is a cohort of older Japanese Americans. These sampling differences have resulted in differences in the age and sex distributions within the cohorts, as well as differences in the level of education of the participants, which may all affect cognitive trajectories.

Furthermore, it is likely that sociodemographic variables that we have not included here, particularly those from mid to late life, may also have a significant influence on people's cognitive aging trajectories. It is also possible that they have a mediating effect on the relationship between childhood deprivation and cognitive aging trajectories.

Finally, it should be noted that membership in the latent trajectory classes are estimated rather than observed, and that the assignment into a class involves a degree of uncertainty. Using these predicted latent trajectory classes can lead to biased estimates for the relationship between the latent classes and the observed predictor (i.e., self‐reported childhood SES in this case).^47^

The primary aim of this study is to explore the relationship between childhood SES and mid‐ to late‐life cognitive change. Using longitudinal data from three cohorts, we characterized latent cognitive trajectories, and examined their association with childhood SES indicators. We found that: (1) there were multiple trajectories for all cognitive outcomes, and (2) lower childhood SES is consistently associated with an increased likelihood of being in a lower cognitive trajectory. These finding indicate that childhood adversity adversely affects cognitive change over time, and not just cognitive performance.

These findings have implications for prevention. Globally, the number of people living with dementia is rapidly increasing, but there is currently no cure and no disease‐modifying treatment. Delaying or preventing the onset of dementia is therefore a key public health priority. Our analyses confirm that early adversity is associated with lower cognitive function and provides evidence that this effect follows through into greater cognitive decline. This suggests that early life experience is an important contributor to an individual's cognitive capital, and that interventions aimed at reducing socioeconomic inequalities may be effective in reducing cognitive inequalities by delaying or slowing cognitive decline.

In summary, different patterns of cognitive decline were observed between cohorts and across cognitive domains, and lower childhood SES generally predicted membership in a lower cognitive trajectory class reflecting more rapid cognitive decline. Future research may benefit from examining trajectories over longer periods, using different cognitive instruments with better psychometric properties, as well as assessing more cognitive domains than examined here.

## CONFLICT OF INTEREST

None.

## Supporting information

SUPPORTING INFORMATION
